# Biodistribution and Clearance of TiO_2_ Nanoparticles in Rats after Intravenous Injection

**DOI:** 10.1371/journal.pone.0124490

**Published:** 2015-04-24

**Authors:** Dan Elgrabli, Remy Beaudouin, Nawel Jbilou, Magali Floriani, Alexandre Pery, Françoise Rogerieux, Ghislaine Lacroix

**Affiliations:** 1 Institut National de l'Environnement Industriel et des Risques (INERIS), Parc technologique Alata, Verneuil en Halatte, France; 2 Laboratoire Matières et systèmes complexes, UMR7057 CNRS/Université paris Diderot, Paris, France; 3 Institut de Radioprotection et de Sûreté Nucléaire Cadarache (IRSN), Saint Paul les Durance, France; Brandeis University, UNITED STATES

## Abstract

Titanium dioxide (TiO_2_) nanoparticles are used in many applications. Due to their small size, easy body penetration and toxicological adverse effects have been suspected. Numerous studies have tried to characterize TiO_2_ translocation after oral, dermal or respiratory exposure. In this study, we focused on TiO_2_ nanoparticle biodistribution, clearance and toxicological effects after intravenous injection, considering TiO_2_ translocation in the blood occurs. Using ICP-OES, transmission electron microscopy, and histological methods, we found TiO_2_ accumulation in liver, lungs and spleen. We estimated TiO_2_ nanoparticles’ half life in the body to about 10 days. Clinical biomarkers were also quantified for 56 days to identify potential toxicological impact on lungs, blood, liver, spleen and kidneys. Results showed absence of toxicological effects after TiO_2_ intravenous injection at concentrations of 7.7 to 9.4 mg/kg.

## Introduction

TiO_2_ nanoparticles (NPs) are used in many applications and found in many products, including paints, coatings, pigments and dyes (57% of the market), plastics (26%), paper (13%). The remaining 4% of TiO_2_ NPs is found in catalytic, cosmetics, ceramics, printing inks, glass, food as an anticaking or whitening agent, etc [1]. Primarily workers, but also general populations are therefore potentially exposed to this nanomaterial either by inhalation, oral or dermal route. Due to NPs small size, easy body penetration and toxicological adverse effects have been suspected [2]. In general, NPs display a greater toxicity than their larger counterparts, including TiO_2_ [3]. Several studies have explored the ability of various TiO_2_ NPs to cross biological barriers such as the lung, gastro-intestinal tract or skin.

In the lungs, a high fraction of NPs, has been found deposited in both tracheobronchial and pulmonary areas [4]. Two studies have shown that nanosized TiO_2_ was less effectively phagocytosed and cleared by alveolar macrophages than fine TiO_2_ particles and thus could enter the alveolar interstitium [5,6]. Once within the alveolar walls, NPs can potentially enter pulmonary capillaries and further translocate to systemic sites. A few studies have addressed this question and showed that NPs can cross the lung barrier. However, the translocation rate requires further investigation. Substantial migration of NPs was found in the liver, heart or blood after inhalation [7–9] whereas a study by Kreyling et al. reported than only 1% of ^192^Ir NPs (15 or 80 nm) after inhalation via an endotracheal tube were found in the liver [10].

Some studies have addressed the consequences of oral exposure to TiO_2_. Distribution and acute toxicity of high dose (5 g/kg) of nano- (25 and 80nm) and micro- (155 nm) sized TiO_2_ were observed after oral exposure [11]. Two weeks after exposure, TiO_2_ was found in lungs, liver, spleen and kidneys, thus providing evidence that TiO_2_ can transfer into the blood from the gastro-intestinal tract. In contrast, although oral TiO_2_ NPs exposure resulted in liver cell degeneration in young and adult rats, no titanium was detected in blood, liver, kidneys and spleen after oral exposure to 200 mg/kg for 30 days. TEM images showed that some particles (size range 60–200 nm) were located in the stomach and small intestine mucosa but no significant translocation in the systemic circulation was reported [12]. Similarly, rats exposed orally to various TiO_2_ NPs (doses: 6.8–8.6 mg/kg or 34–69 mg/kg) showed no or very low titanium levels in liver and spleen and low titanium levels in mesenteric lymph nodes up to 90 days after exposure, suggesting only minor absorption from the gastro-intestinal tract[13].

Several studies have tried to demonstrate the ability of various TiO_2_ NPs differing in their crystalline structure, form, size or formulation to cross the skin barrier. Studies were performed *in vitro* on human, mouse or pig skin sections [14], *in vivo* on human volunteers [14], or on human foreskin grafts transplanted into SCID mice [15]. All these studies reported no TiO_2_ NPs translocation through the skin. Using STIM or PIXE associated with TEM, TiO_2_ particles were mostly detected in the intercellular spaces between the corneocytes of outermost layers of the stratum corneum [14], with presence of marginal TiO_2_ nanoparticles reported at the Stratum Granulosum limit [15]. The potential for TiO_2_ to cause adverse effects depends primarily upon the ability of the nanoparticles to reach viable skin cells and secondly upon the ability to cause damage in vital organs. To date, the current data suggest that TiO_2_ nanoparticles do not reach viable skin cells and remain on the surface and in the outer layer (stratum corneum) of the skin, which is composed of non-viable, keratinized cells. Nevertheless, effects of TiO_2_ in the case of damaged skin and ruptured barrier, remain to be investigated.

In this study, we hypothesized that TiO_2_ NPs could cross biological barriers and reach blood circulation. We investigated biodistribution, target organs and potential adverse effects after intravenous injection. This route of administration allows knowledge of the exact bioavailable dose of TiO_2_ present in rat blood. We applied two kinetic models to analyze the time course of TiO_2_ concentrations in several organs. The first one is a compartmental model, with fixed rates of TiO_2_ transfer from one compartment to another. The second one is a physiologically-based pharmacokinetic (PBPK) model. Such model aims at providing a better description of kinetics, by taking into account the blood perfusion rate and affinity for the substance of interest. In this model, compartments are defined as organs or groups of organs, described by volumes, blood flows, and a coefficient accounting for the partitioning of xenobiotics between blood and organ. Transport between those compartments by blood, lymph, or diffusion, and further absorption, distribution, metabolism or excretion (ADME) processes are described by mathematical equations whose structure is governed by physiology [16,17]. For both models, only organs with significant accumulation throughout time were considered.

## Results

### Characterization of the suspension

**Fig 1:**
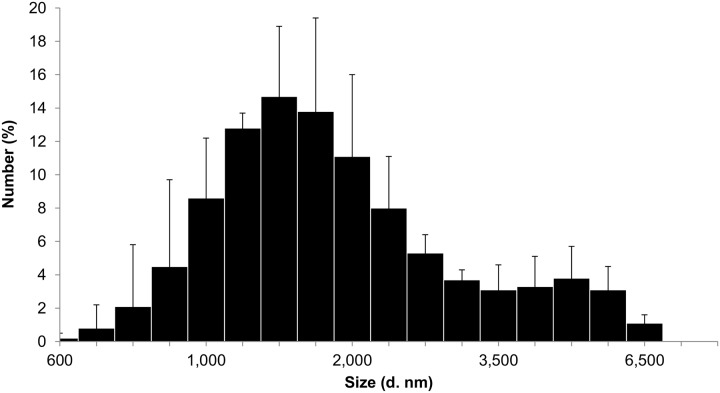
Size distribution of TiO_2_ suspension in NaCl determined by dynamic light scattering.

Size distribution and zeta potential were determined by dynamic light scattering (DLS). DLS measurements showed that TiO_2_ NPs were polydispersed (PdI = 0.29–0.37) and agglomerated with 73.3–95% of agglomerates with a peak size around 1400–1800 nm and 5–26.7% with a peak size around 4000–5000 nm. TiO_2_ NPs have a positive zeta potential in NaCl 0.9% (+ 20 mV) (Fig 1).

### Biodistribution study

The presence of TiO_2_ was assessed by ICP-OES quantification of titanium (Ti) in lungs, liver, kidneys, spleen, brain, lymph nodes (LN), testis, blood and urine of treated and control rats, 10 min, 1h, 1 day, 7, 28 and 56 days after intravenous injection. No increase of Ti was detectable in the brain, lymph nodes, kidneys or testis. A small and short increase of Ti was observed in blood 10 minutes after the injection (0.33 μg versus 0.12 μg for the control) but did not reach statistical significance. A significant increase of Ti was observed in urine 1 day after the injection (1.03 μg versus 0.12 μg for the control) (Table 1). Target organs after intravenous TiO_2_ NPs injection were liver, spleen, and lungs, with the vast majority of injected Ti found in the liver (92.1%), the spleen (3.5%) and the lungs (0.7%) (Table 1). In the lungs, Ti levels were significantly higher than controls at all time points after injection. In the liver and the spleen, Ti levels increased up to 1 day then, decreased regularly 7, 28 and 56 days after the injection, (Table 1).

**Table 1:** Titanium dosage in organs and urine performed by ICP-OES.

μg Ti/Organs	Control	10 mn	1h	1 day	7 days	28 days	56 days
**Kidneys**	**0.95 ± 0.34**	**1.34 ± 0.77**	**0.88 ±0.5**	**1.11± 0.26**	**0.87± 0.26**	**1.29± 0.19**	**1.21± 0.76**
**Brain**	**0.34±0.21**	**0.31±0.20**	**0.30±0.05**	**0.30±0.06**	**0.35± 0.09**	**0.37± 0.17**	**0.29± 0.15**
**Spleen**	**0.42± 0.3**	16.1± 9.05*	25.7±31.9*	35,2± 18.1*	31.7± 5.31*	24.1± 0.31*	13.8± 0.81*
**Lungs**	**0.2 ± 0.1**	5.77±2.32*	**4.81±5.38**	6.89±2.03*	5.59± 1.01*	2.01± 0.14*	7.93± 2.36*
**Liver**	**0.03± 0.01**	637±76*	793±210*	939±160*	813±176*	457±211*	58± 14*
**Blood**	**0.12± 0.05**	**0.33± 0.11**	**0.15± 0.02**	**0.23±0.13**	**0.21± 0.05**	**0.09± 0.05**	**N.D.**
**LN**	**0.24± 0.14**	**0.23± 0.09**	**0.25± 0.13**	**0.16± 0.04**	**0.23± 0.12**	**0.25± 0.07**	**N.D.**
**Testis**	**0.32±0.19**	**0.35± 0.14**	**0.32± 0.06**	**0.21±0.06**	**0.28± 0.09**	**0.28± 0.08**	**0.24± 0.14**
	**Control**	**J1**	**J3**	**J6**	**J27**		
**Urine**	**0.12± 0.03**	1.03± 0.71*	**0.14± 0.11**	**0.33± 0.28**	**0.61± 0.60**		

N.D. Not determined

Each value represents mean from 6 independent experiments ± SD. For each suspension, 3 measurements were performed.

(*) represent a statistical difference (p<0.05).

**Fig 2:**
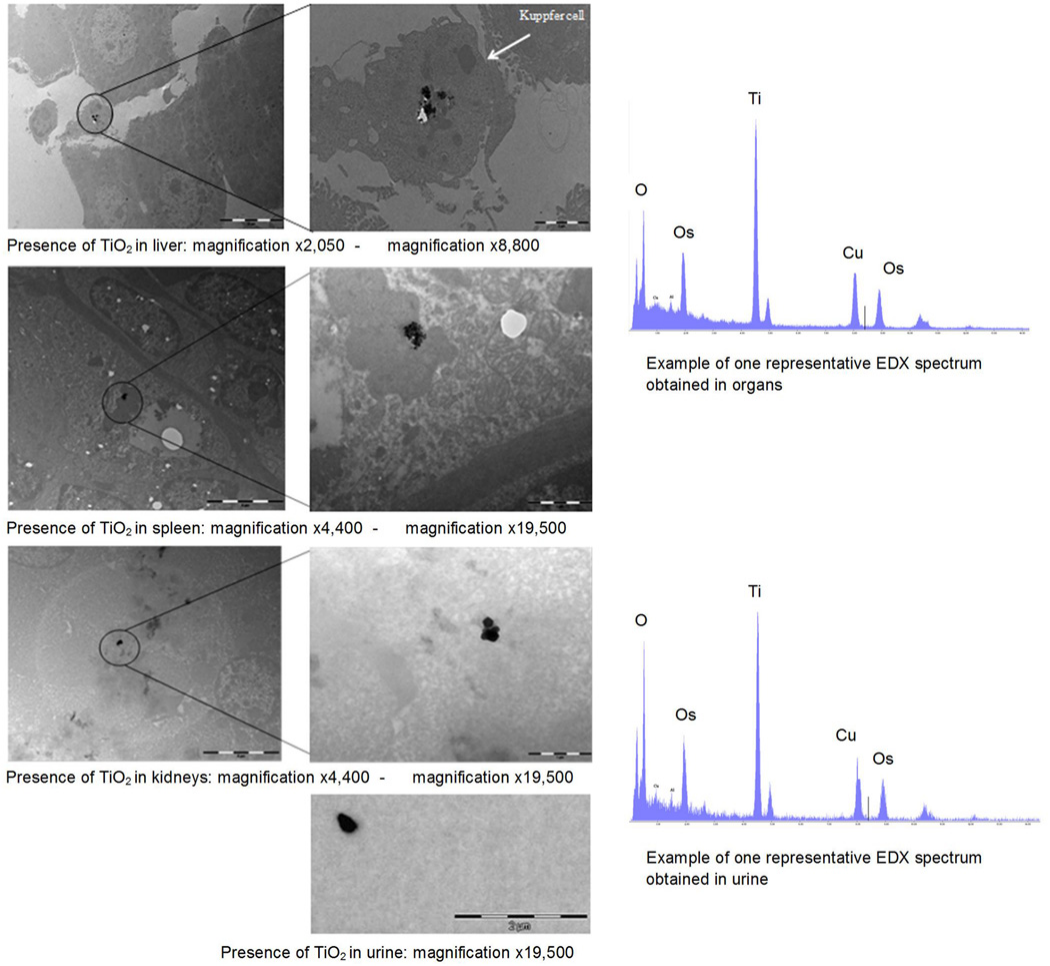
Transmission electron microscopy analysis and energy dispersive X ray microanalysis spectrum of TiO_2_ agglomerates in urine, liver, spleen and kidneys 28 days after intravenous injection.

To confirm the presence of Ti in organs, STEM-EDX analysis of liver, spleen and kidneys of TiO_2_-exposed rats was performed. Ti was observed in urine and in liver, spleen and kidneys (Fig 2).

### Toxicological study

To identify a potential toxic effect of TiO_2_, several toxicological parameters were determined 1, 14, 28 and 56 after the exposure. TiO_2_ exposure did not modify total protein content, IL-1β and IL-6 levels in serum, urea, creatinine, ASAT, proteins, albumin, globulin, γGT, glucose, lactate dehydrogenase, triglycerides, calcium rate, leucocytes, red blood cell, hemoglobin, hematocrit, average blood volume, mean corpuscular hemoglobin, mean corpuscular hemoglobin concentration, blood platelet, basophiles, eosinophiles, neutrophiles, lymphocytes, monocytes, atypical cells, anisocytosis and polychromacytosis. These observations are in favor of a lack of effect of intravenously injected TiO_2_ NPs on heart, spleen, liver, kidneys and overall inflammation.

### Histopathological analysis

**Fig 3:**
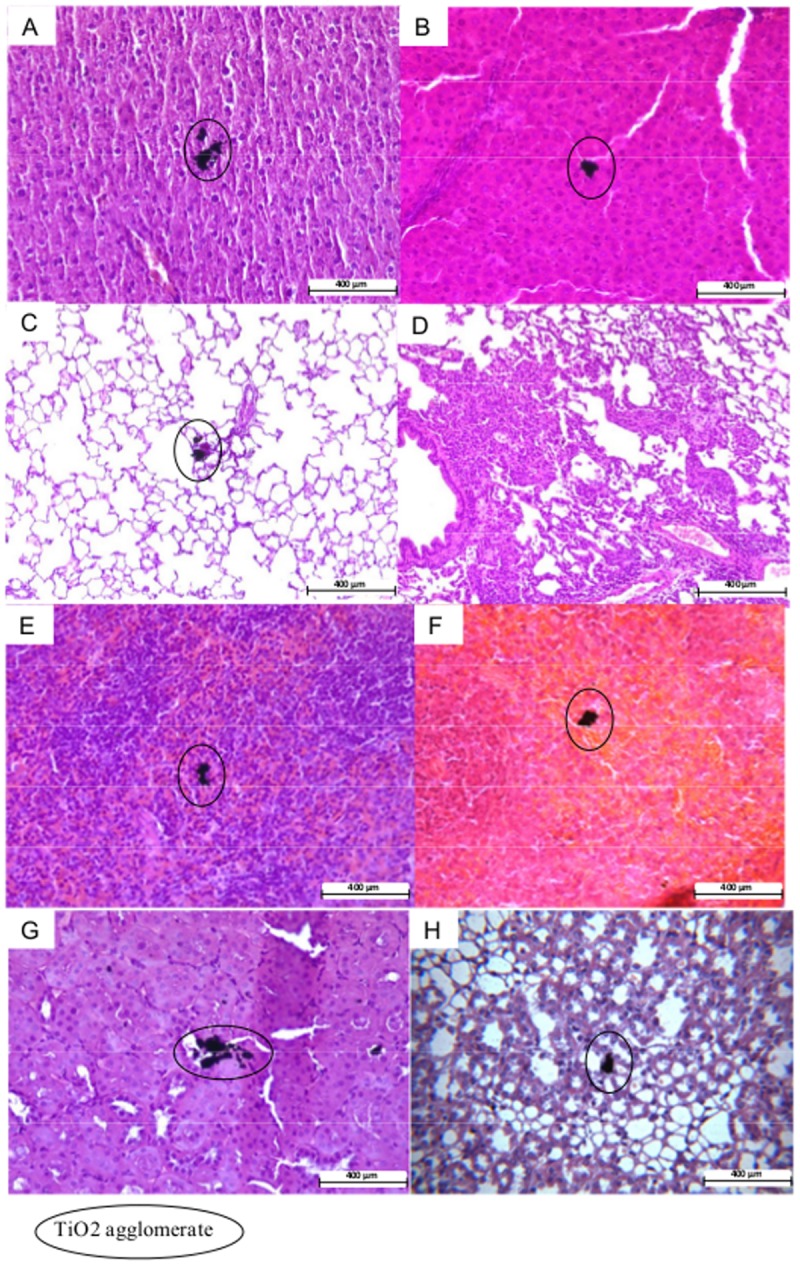
HE optical microscopy analysisof TiO_2_ agglomerates in target organs. (A) Liver, 1 day after treatment, (B) Liver, 56 days after treatment, (C) Lungs, 1 day after treatment, (D) Lungs, 56 days after treatment, (E) Spleen, 1 day after treatment, (F) Spleen, 56 days after treatment, (F) Kidney, 1 day after treatment, (G) Kidney, 56 days after treatment.

Histopathological analysis, using HES and Masson’s trichrome staining of lung, spleen, liver and kidney sections, revealed no significant alterations of the tissue structures (Fig 3). However, TiO_2_ agglomerates were observed in all organs (Fig 3).

### Models to analyze the kinetic data

AIC and BIC values, which describe the ratio between accuracy and complexity of models, confirmed that the compartmental model largely outperformed the PBPK model. Moreover, TiO_2_ kinetics in the lungs could not be described in the PBPK model through partitioning between blood and lungs, due to a longer retention time in lungs compared to blood. We consequently introduced transfer of TiO_2_ from blood to lungs and vice versa in the PBPK model. Finally, estimated values for liver and spleen partition coefficients are particularly unrealistic and obtained in general only for very hydrophobic compounds (such as dioxin), associated with a very low elimination rate, which is not the case here. Consequently, it is very unlikely that TiO_2_ kinetics could be described using a PBPK modeling approach, and therefore a compartmental model is much more appropriate.

**Fig 4:**
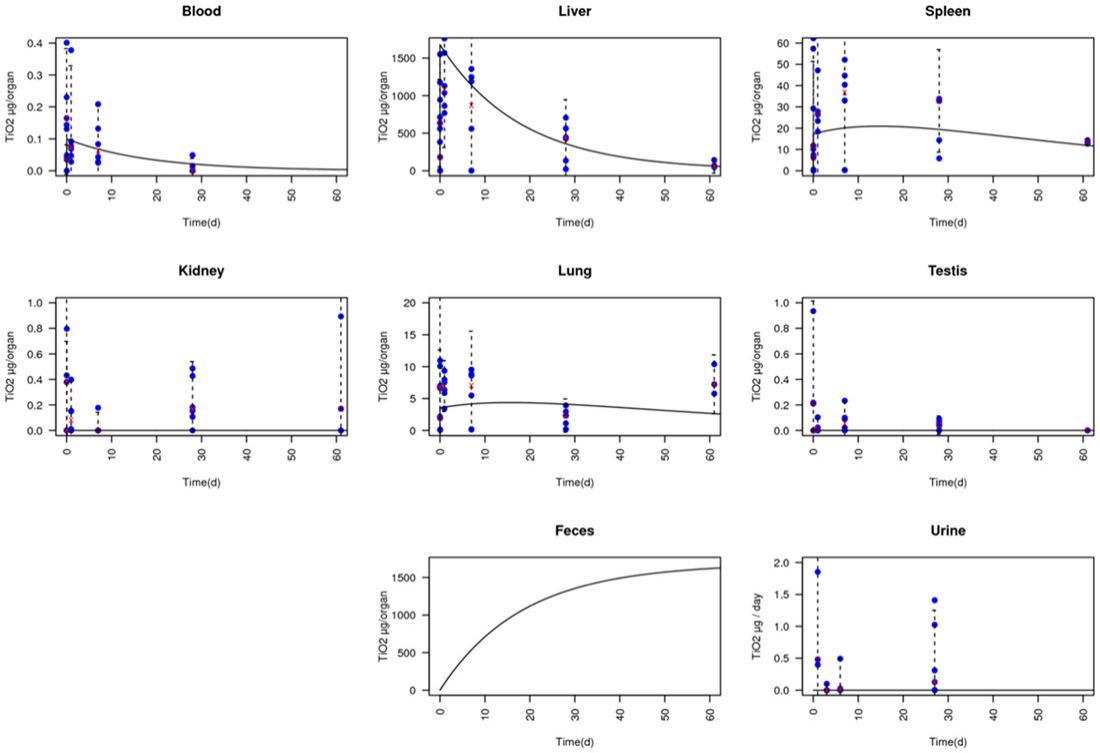
Data correlation in compartmental model. Data are represented as blue crosses. Means of data at each time are represented as pink dots. Confidence interval at 95% is represented by a dotted line (Mean ± 1.96 × SD). Results obtained with the compartmental model are represented as a black line.

Liver is mostly where accumulation occurs. Liver TiO_2_ half-life has been estimated at 12.6 days. The half-life in the body, corresponding to clearance of 50% of the initial amount of TiO_2_, was determined to be 12.7 days. The half-life values in all other organs (50% of the maximum amount of TiO_2_ in organ cleared) are represented on Fig 4. TiO_2_ half-life was determined to be 68.3 days for spleen, 12.56 days for blood and 72.1 days for lungs.

## Discussion

The growing use of TiO_2_ NPs in several industries lead to the question about its toxicity. Many studies tried to identify TiO_2_ capacity to cross biological barriers like skin, gastro-intestinal tract or lungs. Wang *et al*. showed the capacity of TiO_2_ (25, 80 and 155 nm) to cross the gastro-intestinal tract, which was then distributed in lungs, spleen, liver and kidneys two weeks after the exposure [11]. These results are consistent with our data obtained using ICP-OES analysis after intravenous injection. Wang et al. also reported inflammation responses, liver damage including an increase in serum transaminases, hepatic necrosis revealed by histopathological investigations, and increases in cardiac damage markers [11]. In contrast, in our study, no toxicological effects, no tissue damage or general inflammation were observed in liver, kidneys, spleen, heart, after g exposure to 7.7 to 9.4 mg/k TiO_2_ nanoparticles in rats. This discrepancy could be due to the very high dose of TiO_2_ nanoparticles (5g/kg) used in the Wang et al. study. In another study, tissue distribution of TiO_2_ nanoparticles (20–30nm) was assessed in rats following intravenous injection (5mg/kg) 1, 14 and 28 days post exposure [18]. TiO_2_ nanoparticles were cleared from blood and primarily accumulated within liver, with Ti also found in spleen, lungs and kidneys. TiO_2_ levels within the liver were still elevated at the last exposure time point (28 days). However, levels decreased with time in the other organs. Consistent with our study, no serum cytokine or enzyme changes were observed. In a previous study performed by Olemedo et al., the histological analysis of TiO_2_-exposed rats revealed the presence of abundant intracellular aggregates of metallic particles of Ti in peritoneum, liver, lungs and spleen at the higher dose 16,000 mg/kg, 5 month after the injection [19]. Our results are consistent with these observations: liver was also identified as the main target organ after TiO_2_ intravenous injection in rats. Ti was also distributed in spleen, kidney and lung. Depending on the TiO_2_ NPs used, half-lives for liver were estimated between 28–248 days, 45–96 days for lungs, 49–531 days for kidneys and 650 days for spleen (calculated for one TiO_2_ only)[13]. Here, we show similar half-life for TiO_2_ in lungs (72 days). For liver, we estimated the half-life to be 12.6 days, which is shorter than the half-life of the previous study. However, this discrepancy may be explained by the different TiO_2_ NPs used between both studies.

Kinetic models, such as PBPK models should be further developed to allow better interpretation of nanotoxicity data, guide in vivo study design, and accelerate nanoparticle risk assessment [20], in particular because these models allow to integrate multiple information obtained in vitro on cells. However, due to the differences between nanoparticles and small molecules, modifications are needed to build appropriate PBPK models for nanoparticles [21]. In fact, few physiologically based PBPK models were developed to predict the kinetic of nanoparticles [22]. However, if PBPK models developed for other nanoparticle than TiO_2_ could be considered appropriate [23,24], in our study, compartmental model was more adequate because TiO_2_ toxicokinetic is not simply related to the blood flows. The concept of partitioning between tissues and blood is clear for conventional substances and is based on the chemical potential of molecules in different phases such as water, fat and protein phase. It is less clear what determines the partition of nanoparticles [23,24]. A more complex PBPK model with more parameters including either transport limited kinetics or diffusion limited kinetics could better fit the data [23]. Nevertheless, to develop this kind of model, more detailed data need to be acquired. Here, a compartmental model outperformed PBPK modeling, suggesting that distribution through blood and partitioning between blood and organs would be a poor concept to understand the kinetics of nanoparticles.

In conclusion, we showed that TiO_2_ nanoparticles were quickly eliminated from blood and relocated in liver, spleen and lungs. Here, we showed that TiO_2_ nanoparticles could be cleared from the body with a half-life of 12.7 days.

## Materials and Methods

### Nanoparticles

Anatase TiO_2_ nanoparticles were purchased from Sigma-Aldrich (Lyon, France), reference 637254. Their diameter is below 25 nm and the density is 3.9 g/ml. Fusion temperature was determined as 1825°C and specific surface area as 44–55 m^2^/g (supplier data).

### Preparation and characterization of TiO_2_ suspensions

TiO_2_ nanoparticles were suspended in NaCl 0.9% at a concentration of 5 mg/ml. Homogeneity of the suspensions were obtained after 15 mn sonication in ultrasonic bath (Elmasonic S 30H, Elma) and 10s vortex. The size distribution and zeta potential were determined by dynamic light scattering (DLS, Zetasizer nano ZS, Malvern). As a concentration of 5 mg/ml was too high for DLS measurements, suspension at 1 mg/ml was used for assessment of size distribution and zeta potential.

### Animals

Male Sprague-Dawley rats weighing 180–220 g (6–7 week-old) were purchased from Charles River Laboratories (St Germain-sur-l’Arbresle, France). Rats were kept in a conventional animal facility and had access to food and drink *ad libitum*. The experimental protocol was approved by National Institute of Industrial Environment and Risks (INERIS) internal ethical committee for animal research.

### Biodistribution study

Rats were treated with intravenous injection of 1.7 mg TiO_2_/rat (333 μl of 5mg/ml suspension) or with 333 μl of NaCl 0.9% suspension as control and sacrificed 10 mn, 1h, 1, 7, 28, and 56 days later. For each recovery period, 6 rats were used. Control and exposed animals were sacrificed by intraperitoneal injection of pentobarbital (150 mg/kg) and lungs, liver, kidneys, spleen, brain, lymph nodes, testis and blood were harvested for ICP-OES Ti assay. Rats sacrificed at 56 days were kept in metabolic cages for 24h before every sacrifice to collect urine and feces at J1, J3, J6 and J27 for ICP-OES analysis.

### ICP-OES assay

The organs and urine were incinerated in an oven for 12h at 120°C and 12h at 600°C. Ashes were suspended in 9.9 ml distilled water and 0.1 ml nitric acid. Samples were analyzed for Ti content by ICP-OES (Ultima, HORIBA Jobin Yvon, Edison, NJ) after 15 mn sonication in ultrasonic bath (Elmasonic S 30H, Elma) and 10s vortex.

### Transmission Electron Microscopy (TEM) and TiO_2_ analysis by Scanning and Energy Dispersive X ray microanalysis (STEM-EDX)

Liver, kidneys and spleen were removed, cut in small sections and fixed with 2.5% glutaraldehyde. After a postfixation with 1% osmium tetroxide, samples were dehydrated by ethanol and embedded in EPON 812 (TAAB). Ultrathin sections of 90 nm and 150 nm respectively for TEM and STEM-EDX analysis were obtained by an ultramicrotome (UCT, Leica), mounted on copper grids and stained with uranyl acetate and examined in a Tecnai G2 Biotwin (FEI) electron microscope using an accelerated voltage of 100 kV. Several photographs of entire cells and local detailed structures were taken, analysed and compared to NaCl control samples.

### Toxicological study

Liver, spleen and kidney functions as well as general inflammation were assessed by measuring various blood biomarkers. At sacrifice, 2 ml of blood from control and treated rats were collected after 1, 14, 28 and 56 days. To minimize impact of food on biomarker levels, rats were fasted 12h before blood sampling. Organ function and inflammation were assessed using biochemical dosages in serum (urea, creatinine, ASAT, proteins, albumin, globulin, γGT, glucose, lactate dehydrogenase, triglycerides and calcium) and by hematological biomarkers quantification (leucocytes, red blood cell, hemoglobin, hematocrit, average blood volume, mean corpuscular hemoglobin, mean corpuscular hemoglobin concentration, blood platelet, basophiles, eosinophiles, neutrophiles, lymphocytes, monocytes, atypical cells, anisocytosis and polychromacytosis). All dosages were performed in accordance with clinical norms by Idexx (Germany), an expert laboratory dedicated to veterinary for clinical studies.

Inflammation was also studied with cytokine dosage in blood. Total protein content was assessed using Bradford method [25]. Specific protein quantifications (IL-1β and IL-6) were performed with ELISA kits (Duoset, R&D Systems) according to the manufacturer’s instructions.

### Histopathological analysis

Tissue specimens from lungs, liver, kidneys and spleen were fixed in 10% formaldehyde and processed for paraffin embedding. Tissue sections (5 μm) were stained with hematoxylin, eosin and saffron (HES) and with Masson’s trichrome to examine general organs morphology.

### Models to analyze the kinetic data

**Fig 5:**
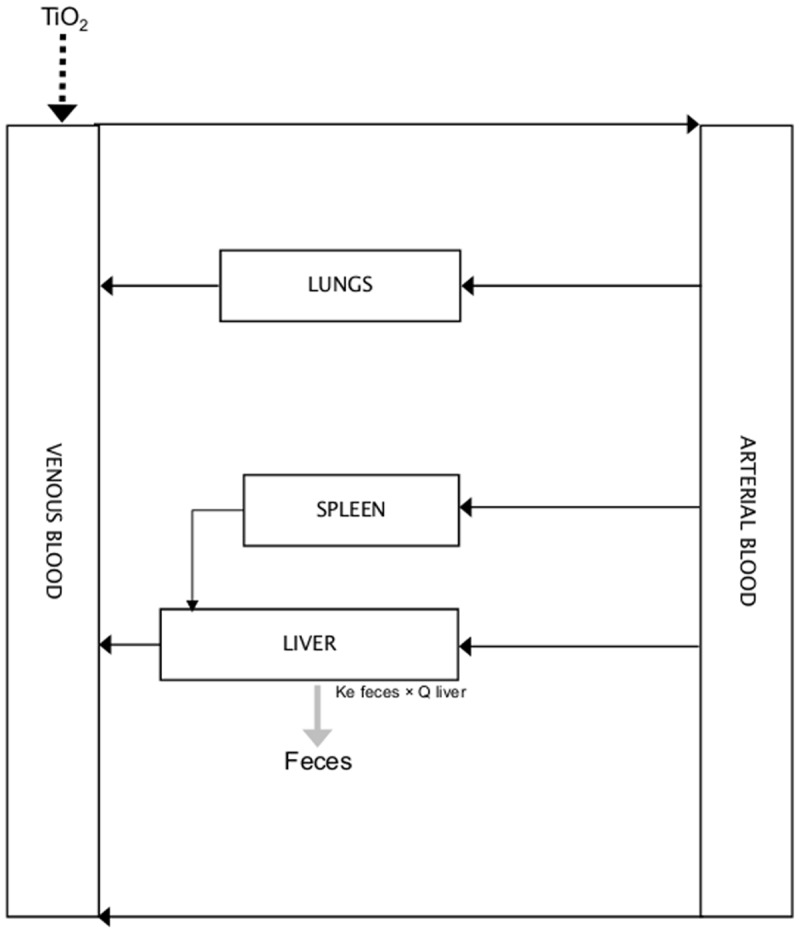
Schematic representation of the kinetic models.

We tested two different models, a compartmental one and a PBPK one. The selected compartments were the same for both models (Fig 5). Only the ones with significant accumulation compared to control data were selected. As urine data showed little recovery of initial TiO_2_, we considered that biliary elimination from liver was the elimination route to account for in the model. This elimination has a rate proportional to liver quantity of TiO_2_. Prior to analysis, background of TiO_2_ concentrations (mean measures in control) were removed to the measured concentrations for exposed rats.

The parameters of the models are provided in Table 2

**Table 2:** Parameters used in the two kinetic models.

Compartmental model	Transfer rate from blood to spleen	Kd_blood_spleen (d^-1^)	9.6994
	Transfer rate from blood to liver	Kd_blood_liver (d^-1^)	946.12
	Transfer rate from blood to lungs	Kd_blood_lungs (d^-1^)	1.9863
	Transfer rate from spleen to liver	Kd_spleen_liver (d^-1^)	0.02068
	Transfer rate from liver to blood	Kd_liver_blood (d^-1^)	0.0569
	Transfer rate from lungs to blood	Kd_lungs_blood (d^-1^)	0.0191
	Transfer rate from liver to bile	Kd_liver_bile (d^-1^)	0.0551
PBPK model	Body weight	BDW (g)	250
	Liver weight	scV_li (portion of BDW)	0.04
	Blood weight	scV_b (portion of BDW)	0.074
	Spleen weight	scV_s (portion of BDW)	0.00267
	Lung weight	scV_lu (portion of BDW)	0.005
	Cardiac output	Q_card (mL/d/g)	633
	Liver blood flow	scQ_li (portion of BDW)	0.177
	Spleen blood flow	scQ_s (portion of BDW)	0.006
	Lung blood flow	scQ_lu (portion of BDW)	0.021
	Partition coefficient between blood and liver	PC_liver	78 000
	Partition coefficient between blood and spleen	PC_spleen	58 100
	Transfer rate from blood to lungs	Kd_blood_lung (d^-1^)	3.12
	Transfer rate from lungs to blood	Kd_lungs_blood (d^-1^)	1.85e-04
	Transfer rate from liver to bile	Kd_liver_bile (d^-1^)	1.91

Physiological parameters used in the two kinetic models are from Brown et al., 1997[27] and Haddad et al., 1998[28]. Other parameters have been estimated in the present study.

In the compartmental model, the contribution to respective loss and gain of TiO_2_ in compartments i and j due to transfer from compartment i to compartment j equals Ki-jQi where Ki-j is the transfer rate and Qi the quantity of TiO_2_ in compartment i.

In the PBPK model, the concentrations in organ i was described by the following equation:

$$\frac{{\text{dC}}_{\text{i}}}{\text{dt}}={\text{Q}}_{\text{i}}\left({\text{C}}_{\text{b}}-\frac{{\text{C}}_{\text{i}}}{{\text{PC}}_{\text{i}}}\right)$$

Where Qi is blood flow in organ i, Ci the concentration in the organ i, Cb the concentration in the blood entering the organ and PCi the partition coefficient of the organ i.

### Statistics

All data were considered normally distributed and expressed as mean ± S.D (standard deviation). F-test was used to compare the homogeneity of the variances. If homogeneity of variance was verified with a risk alpha equal to 5%, differences between each group were assessed with a one-way analysis of variance (ANOVA). When all ANOVA tests were positive, groups were subjected to the multiple comparisons Dunnett's test. If variances were not homogeneous, each group was compared to control with the Mann-Whitney test. *P < 0.05 was considered as the statistical significance level.

Parameters of the kinetic models were estimated through maximum likelihood methods. The measurement errors were assumed to be independent and log-normally distributed, with a geometric mean equal to the model predictions and a geometric standard deviation (GSD) of 1.1 (approximately 10% error). Data likelihoods were therefore given by:

$$\text{log}\left(Y\right)\sim N\left(\log\left(F\left(X,\theta \right)\right),\sigma_{c}\right)$$

where the function F(X,θ) corresponds to a PBTK model with input X and parameters θ, and σc is equal to 1.1. Parameters estimation and building of confidence intervals were performed using the MCSim software[26].

The performances of the compartmental and of the PBPK model were compared through AIC (Akaike Information Criteria) and BIC (Bayesian Information Criteria) calculations. Both criteria describe the trade-off between accuracy and complexity (number of parameters) of models. BIC is closely related to AIC, but the penalty term is larger in BIC than in AIC.

AIC=2k−2.ln(L)

BIC=−2.ln(L)+k.ln(n)

Where k is the number of parameters in the model, L is for likelihood and n the number of datapoints.
